# Sediment and Turbidity Associated with Offshore Dredging Increase Coral Disease Prevalence on Nearby Reefs

**DOI:** 10.1371/journal.pone.0102498

**Published:** 2014-07-16

**Authors:** F. Joseph Pollock, Joleah B. Lamb, Stuart N. Field, Scott F. Heron, Britta Schaffelke, George Shedrawi, David G. Bourne, Bette L. Willis

**Affiliations:** 1 ARC Centre of Excellence for Coral Reef Studies, School of Marine and Tropical Biology, James Cook University, Townsville, Queensland, Australia; 2 AIMS@JCU, Australian Institute of Marine Science and James Cook University, Townsville, Queensland, Australia; 3 Australian Institute of Marine Science, PMB 3, Townsville, Queensland, Australia; 4 Department of Parks and Wildlife, Marine Science Program, Kensington, Western Australia, Australia; 5 Oceans Institute, University of Western Australia, Crawley, Western Australia, Australia; 6 NOAA Coral Reef Watch, Townsville, Queensland, Australia; 7 Marine Geophysical Laboratory, School of Engineering and Physical Sciences, James Cook University, Townsville, Queensland, Australia; The Australian National University, Australia

## Abstract

In recent decades, coral reef ecosystems have declined to the extent that reefs are now threatened globally. While many water quality parameters have been proposed to contribute to reef declines, little evidence exists conclusively linking specific water quality parameters with increased disease prevalence *in situ*. Here we report evidence from *in situ* coral health surveys confirming that chronic exposure to dredging-associated sediment plumes significantly increase the prevalence of white syndromes, a devastating group of globally important coral diseases. Coral health surveys were conducted along a dredging-associated sediment plume gradient to assess the relationship between sedimentation, turbidity and coral health. Reefs exposed to the highest number of days under the sediment plume (296 to 347 days) had two-fold higher levels of disease, largely driven by a 2.5-fold increase in white syndromes, and a six-fold increase in other signs of compromised coral health relative to reefs with little or no plume exposure (0 to 9 days). Multivariate modeling and ordination incorporating sediment exposure level, coral community composition and cover, predation and multiple thermal stress indices provided further confirmation that sediment plume exposure level was the main driver of elevated disease and other compromised coral health indicators. This study provides the first evidence linking dredging-associated sedimentation and turbidity with elevated coral disease prevalence *in situ*. Our results may help to explain observed increases in global coral disease prevalence in recent decades and suggest that minimizing sedimentation and turbidity associated with coastal development will provide an important management tool for controlling coral disease epizootics.

## Introduction

Over the last 30 years, hard coral cover has decreased by an average of 50% on Indo-Pacific reefs and 80% on Caribbean reefs [1]–[3]. While these declines have been attributed to a number of factors, including water pollution, habitat destruction, overfishing, invasive species, and global climate change [1], [2], coral diseases have recently emerged as a significant driver of global coral reef decline [4], [5]. The destructive potential of coral disease is most clearly exemplified in the Caribbean where successive disease outbreaks have decreased acroporid cover by up to 95% and contributed substantially to observed ecological phase shifts from coral to algal-dominated reefs [6]–[8]. Furthermore, the incidence of coral epizootics has increased globally over the last 20 years [3], [6], [9], [10], highlighting the need to understand and manage the factors underlying coral disease outbreaks.

Reduced water quality caused by explosive human population growth is often cited as an important factor driving coral disease epizootics [11]–[13]. Land clearing exposes 1% of the Earth's surface to eroding processes annually and urbanization of coastal areas is expanding disproportionally to population growth [14], [15]. Consequently, coastal coral reefs, like many other marine ecosystems, are increasingly subjected to elevated levels of eutrophication, sedimentation and turbidity, factors proposed to compromise disease resistance of corals and/or increase pathogen virulence [13], [16]. Coastal dredging for land reclamation, beach nourishment and port construction further exacerbates terrestrial nutrient and sediment influx by resuspending benthic sediments [17]. Additionally, more frequent and intense storms associated with climate change amplify water quality declines by promoting coastal runoff and sediment resuspension [18]. Eutrophication, and more specifically nutrient enrichment, has been shown to exacerbate existing coral disease infections and shift coral-associated microbial communities towards communities typical of diseased corals, but little is known about the role of nutrients in disease initiation [13], [19], [20].

Sedimentation and turbidity, associated with both weather events and anthropogenic activities, are also frequently proposed to contribute to increased coral disease prevalence [16], although empirical evidence is lacking. Hodgson [21] suggested sedimentation as a potential mechanism for the transmission of coral pathogens from marine or terrestrial substrates onto nearby corals. Silt-associated bacteria were identified as a possible cause of necrosis in sediment-damaged corals, since antibiotic-treated water reduced tissue damage in experimentally silted corals. In field-based observations, Haapklyä et al. [22] noted a correlation between seasonal coastal runoff, including increased sedimentation and turbidity, and the prevalence of coral disease on inshore reefs. Elevated turbidity reduces the amount and quality of ambient light available for photosynthesis by the corals' endosymbiotic algae (*Symbiodinium*) and excess sedimentation inhibits the heterotrophic feeding efficiency of corals, reducing the energy intake of both symbiotic and asymbiotic corals [23], [24]. While corals are able to shed some sediment through mucus production and ciliary action, these mechanisms are energetically expensive and further burden the corals' already reduced energy budgets [25], [26]. Despite a wealth of circumstantial evidence indicating sedimentation and turbidity as potential coral disease drivers [15], [22], [27], no studies have directly linked sedimentation, turbidity and coral disease in the field. Given that nearly 40% of coral reefs are located adjacent to large population centers and coastlines under rapid development to accommodate expanding urban activities [28], effective coastal management will increasingly depend upon a comprehensive understanding of the impacts of sediment, turbidity and associated water quality decline, on all aspects of coral reef health.

Here, we describe the first study to examine the influence of elevated sedimentation and turbidity on coral disease levels *in situ*. We performed detailed coral health assessments along a gradient of exposure to a sediment-laden dredge plume within the Montebello and Barrow Islands off the northern coast of Western Australia. The otherwise relatively pristine conditions of these offshore reefs enabled an empirical examination of the relationship between sedimentation, turbidity and coral disease prevalence in the absence of other confounding influences. Our results indicate that elevated sedimentation and turbidity can significantly increase coral disease prevalence and highlight the urgent need to manage coastal development near coral reef ecosystems.

## Methods

### Ethics statement

This research was conducted under the following permits: Western Australia Department of Environment and Conservation Collection Permit number SF008340 and Western Australia Department of Environment and Conservation Export Permit number ES002169.

### Study site

**Figure 1:**
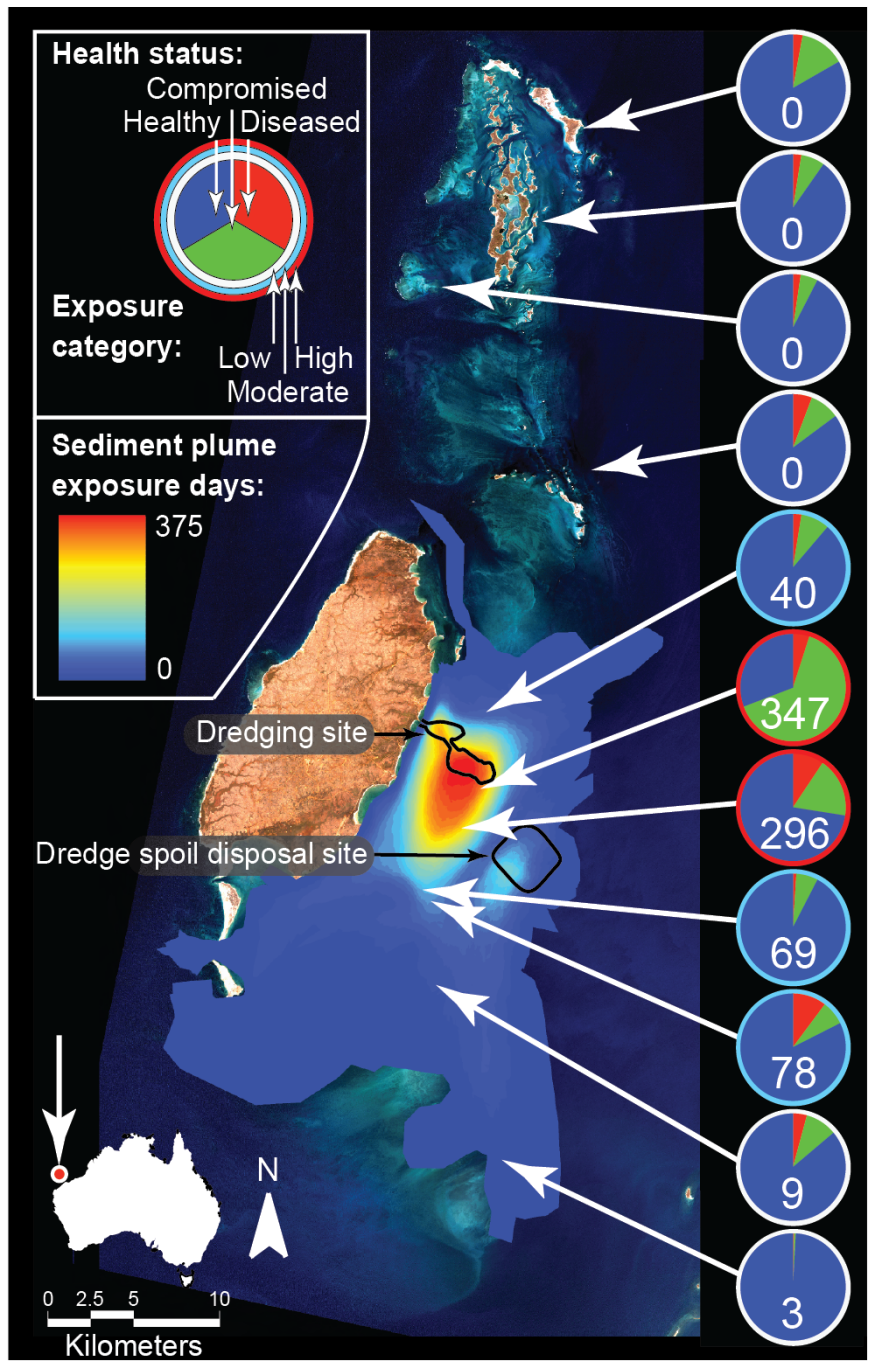
Map showing study site and coral health survey locations at Montebello and Barrow Islands, Western Australia. Colored overlays (gradient from red to blue) indicate satellite-derived sediment plume exposure days determined by hot spot analysis of MODIS satellite imagery. Pie charts indicate the proportion of colonies at each site (n = 3 transects per site) recorded as apparently healthy (blue), diseased (red) or displaying other signs of compromised coral health (green). Numbers inset on pie charts indicate satellite-derived sediment plume exposure days at each site. Colors ringing pie charts indicate plume exposure categories, i.e. white: low (0 to 9 exposure days); blue: moderate (40 to 78 exposure day); and red: high (296 to 347 exposure days).

The Montebello and Barrow Islands are situated in the Pilbara region of Northwest Australia, approximately 1,600 km north of Perth (see Figure 1). The Montebello and Barrow Islands Marine Protected Areas (MBIMPA), incorporating the Montebello Islands Marine Park, Barrow Island Marine Park and the Barrow Island Marine Management Area, were gazetted in 2004. The environment within the MBIMPA is considered to be relatively pristine as a consequence of low human usage, minimal terrestrial influence and strict management controls on industrial developments in the area [29].

The Gorgon Project, based on Barrow Island (20.80°S, 115.40°E), is one of the world's largest natural gas projects and the largest single resource natural gas project in Australia's history. The Gorgon Project dredging program involved the removal and dumping of approximately 7.6 million tons of marine sediment over an 18-month period from 19 May 2010 to 7 November 2011.

### Satellite-derived assessment of sediment plume extent

The area affected by the dredging-induced sediment plume area was quantified daily over the duration of the dredging program using Moderate Resolution Imaging Spectroradiometer (MODIS) satellite imagery, as described by Evans et al. [30]. Briefly, the sediment plume boundary was interpreted manually using one of up to two MODIS images captured daily. A single practitioner manually assessed plumes visible in MODIS images and distinguished these sediment plumes from benthic substrates. Quality control measures were utilized to ensure the collection of consistent, high-quality data (see Evans et al. [30] for details). A ‘hotspot’ analysis was performed on the cumulative daily plume boundaries, for the total number of days available, to provide a dataset describing the number of days the sediment plume was present at any position within the waters surrounding the Montebello and Barrow Islands. These data were used to determine sediment plume exposure days, which are defined here as the cumulative number of days a suspended sediment plume was visible in satellite images at a given location throughout the duration of dredging operations. One year of pre-dredging MODIS imagery was also analyzed to identify a baseline for naturally occurring turbidity events.

### Coral health and community composition surveys

Coral health surveys were conducted in December 2011, one month after the completion of the 18-month Gorgon Project dredging program. Eleven sites were selected, extending both north and south from the dredging site, representing a gradient of sediment plume exposure (Figure 1). At each site, three 15 m × 2 m belt transects, placed haphazardly at least 5 m apart, were surveyed along depth contours at 2 to 6 m, consistent with standardized protocols developed by the Global Environment Facility and World Bank Coral Disease Working Group [31]. Within each 30 m^2^ belt transect, every scleractinian coral colony over 5 cm in diameter was identified to genus-level and classified as either diseased [i.e., affected by one or more of the following diseases: white syndromes (WS), brown band disease, skeletal eroding band, black band disease, and/or growth anomalies]; showing other signs of compromised health (i.e., tissue necrosis associated with sediment accumulation, bleaching, pigmentation response, and/or sponge, red algae, or green algal overgrowth); or healthy (i.e., no visible signs of disease lesions or other indicators of compromised health) using indicators described by Willis et al. [32]. Additionally, signs of coral predation by *Drupella sp*. and/or *Acanthaster planci* (crown-of-thorns seastar; COTS) were recorded. Standard line-intercept surveys were used to determine coral cover and coral community composition to the genus-level by estimating the linear extent of each coral to the nearest centimeter along the central line of each 15 m belt transect. These protocols allow the data collected in this study to be directly compared to other similar standardized coral disease datasets worldwide.

### Assessment of temperature-based risk of disease

Relationships between diseases and anomalously warm temperature have been determined for various marine and terrestrial organisms [15]. To evaluate the role that thermal stress might have played in shaping the spatial patterns of coral disease and other signs of compromised coral health observed, we analyzed temperature-based predictors of disease outbreak risk based on published empirical relationships between temperature metrics, coral cover and disease abundance (summarized in Heron et al. [33]). While these temperature-disease relationships were derived for only one disease type (WS) affecting one coral genus (*Acropora* spp.) on the Great Barrier Reef, the thermal stress predictors of disease risk provide useful indicators of host susceptibility and potentially pathogen loads [33]. Briefly, retrospective Pathfinder satellite sea-surface temperature (SST) time-series for the period 1985 to 2009 were concatenated with NOAA's near real-time 11 km SST time-series (February 2009 to December 2011); concatenation was performed by bias-adjusting the latter dataset to match the former based on the overlap period. The resulting weekly dataset provided a SST time-series for each survey location throughout the dredging period (May 2010 to November 2011) and an internally consistent climatological baseline for the calculation of thermal stress metrics. Five temperature-based stress metrics associated with disease likelihood were derived (see Table 1 for definitions): Hot Snap, Cold Snap and Winter Conditions (see [34] for full details); and mean positive summer anomaly (MPSA) and predicted abundance [35]. Predicted abundance of disease cases per 1,500 m^2^ (*A_disease_*) was calculated using MPSA and total hard coral cover for all species (TCC) from the field surveys, following the model of Maynard et al. [35];
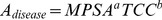
(1)where *a* = 1.07 and *b* = 1.59 [37]. All temperature-based metrics were assessed at the site level (i.e., no replication at the transect level) due to the limited resolution of satellite-derived SST data.

**Table 1:** Environmental predictor variables assessed in a multivariate multiple regression analysis (DISTLM) at sites within three sediment plume exposure categories determined by MODIS satellite imagery: low (0 to 9 plume exposure days, n = 6 sites), moderate (40 to 68 plume exposure days, n = 3 sites) and high (296 to 347 plume exposure days, n = 2 sites); and results of an analysis of variance (ANOVA) for each predictor variable among dredge plume exposure groups.

Factor		Sediment plume Exposure Category					
		Low (n = 6 sites)	Moderate (n = 3 sites)	High (n = 2 sites)	ANOVA	Assessed in	
		mean (SE)	mean (SE)	mean (SE)	F_(2,11)_, *p*	DISTLM?	Source
Dredging:	Sediment plume exposure^a^ days^a^	2.0 (1.5)	62.3 (11.5)	321.5 (25.5)	285.7, <0.001*	Yes	[30]
Predation:	COTS scars^b^	0.0 (0.0)	1.6 (1.1)	0.0 (0.0)	1.5, 0.29	Yes	This study
	*Drupella* scars^c^	3.6 (0.6)	11.7 (3.3)	1.4 (0.9)	2.9, 0.11	Yes	This study
Coral cover:	Total hard coral cover^d^	36.0 (15.0)	53.5 (21.1)	26.0 (20.4)	1.7, 0.24	Yes	This study
Thermal stress:	Peak SST^e^	30.7 (0.2)	30.8 (0.3)	30.9 (0.0)	0.2, 0.81	No^k^	[34]
	Peak SSTA^f^	2.8 (0.1)	2.7 (0.2)	2.7 (0.0)	0.07, 0.94	No^k^	[34]
	Hot Snap^g^	2.0 (1.7)	1.8 (1.4)	2.2 (0.4)	0.04, 0.96	Yes^k^	[34]
	Winter Conditions^h^	10.3 (0.7)	9.0 (0.1)	8.0 (0.2)	2.2, 0.18	Yes	[34]
	MPSA^i^	0.3 (0.1)	0.2 (0.03)	0.3 (0.01)	1.2, 0.35	Yes	[35]
Modeled disease:	Predicted disease abundance^j^	101 (94)	88 (39)	52 (57)	0.3, 0.75	No	[35]

a Number of days the sediment plume was recorded over a site for the duration of dredging operations (days).

b Prevalence of coral colonies with crown-of-thorns seastar lesions determined by *in situ* coral health surveys (%).

c Prevalence of coral colonies with *Drupella* lesions determined by *in situ* coral health surveys (%).

d Total hard coral cover on transects determined by line intercept method (%).

e Maximum sea surface temperature recorded during dredging operations (May 2010 to November 2011) (°C).

f Maximum excursion of sea surface temperature from the long-term climatological value during dredging operations (May 2010 to November 2011) (°C).

g Accumulation of thermal anomalies greater than the long-term summer mean temperature plus one standard deviation (°C-weeks).

h Accumulation of winter anomalies (+ and −) from the long-term winter mean temperature (°C-weeks).

i Average of summer temperature anomalies greater than zero calculated from the monthly mean temperature plus one monthly standard deviation.

j Modeled numeric prediction of disease abundance based upon MPSA and total coral cover (disease cases per 1,500 m^2^).

k Peak SST and Peak SSTA were excluded from the DISTLM due to a strong correlation with Hot Snap (r = 0.87 and 0.79, respectively).

### Data analyses

Prevalence values for coral diseases and other signs of compromised health were calculated within each 30 m^2^ belt transect by dividing the number of colonies with signs of any of 5 diseases recorded or of 6 other indicators of compromised health by the total number of colonies present. To assess the effect of dredging on disease prevalence and on other indicators of compromised health along the plume gradient, sites were assigned to one of three exposure categories based on the number of days a dredging-associated suspended sediment plume was visible in usable MODIS satellite images (Figure 1):

- low-exposure (0–9 sediment plume exposure days; 18 transects),
- moderate-exposure (40–78 sediment plume exposure days; 9 transects) and
- high-exposure (296–347 sediment plume exposure days; 6 transects).

To analyze patterns of coral disease and other signs of compromised health among broad coral growth forms within each sediment plume exposure category, coral genera were assigned to one of three growth form categories: massive, plating or branching (see Methods S1 in File S1). Associations between the prevalence of disease and other compromised coral health indicators and sediment plume exposure days were tested with Pearson product-moment correlations. Differences in mean prevalence levels among the three sediment plume exposure groups were analyzed using two-way (sediment plume exposure category, site) nested analyses of variance (ANOVA), with site treated as a random factor that was nested within the fixed factor, plume exposure. Plume exposure days, coral predation by COTS and *Drupella*, and total hard coral cover were compared among plume exposure groups using the two-way ANOVA design described above. Differences in mean prevalence of disease and compromised health indicators were compared among growth forms within each dredge exposure category using one-way ANOVAs. Similarly, all temperature-based measures of disease likelihood were compared using one-way ANOVAs. Associations between sediment-exposure days and prevalence of coral health and disease were tested with Pearson product-moment correlations. Prior to analyses, assumptions of normality (Shapiro-Wilks) and homogeneity of variance (Levene's test of homogeneity) were tested. Post-hoc comparisons between groups were performed using Tukey's HSD tests. All univariate statistical analyses were performed using STATISTICA 10 (StatSoft, Tulsa, Oklahoma).

Coral community composition (at the genus-level) was compared among reefs within the three sediment plume exposure categories to ensure that community structure was homogenous across treatments. To test for differences in community composition, a nested permutational analysis of variance (PERMANOVA) was used, in which site was treated as a random factor nested within the fixed factor, plume exposure [36]. Similarities among coral communities were illustrated using a non-metric multidimensional scaling plot (nMDS) [36].

A non-parametric distance-based linear model (DISTLM) was used in combination with distance-based redundancy ordination analysis (dbRDA) to explore the hypotheses that variability in patterns of disease and other compromised health indicators could be explained by environmental variables known to impact coral condition and distribution (i.e., sediment plume exposure days, hard coral cover, predation, and calculated thermal stress; see Table 1). The DISTLM models the relationship between these environmental variables and the multivariate coral health prevalence dataset based on a multiple regression model. This routine finds the linear combination of variables that explains the greatest amount of variation in the coral health dataset and examines the amount of variance explained by each environmental variable, providing a pseudo-F statistic. The best-fit model, based on corrected Akaike's Information Criterion (AICc), was then visualized in multidimensional space using dbRDA ordination [36]. Preliminary diagnostics to assess multi-collinearity among predictor variables using draftsman plots reveal two thermal stress indicators, Peak SST and Peak SSTA, were highly correlated with Hot Snap (r = 0.87 and 0.79, respectively). To avoid redundancy, Peak SST and Peak SSTA were not included in the DISTLM or dbRDA. Predictors that best explained the data were overlaid as biplots representing the strength (vector length) and direction of influence [36]. All multivariate analyses were conducted in PRIMER v6 [37] and PERMANOVA+ [36] using Bray-Curtis similarity matrices based on fourth-root transformed data.

## Results

### Satellite-derived assessment of sediment plume extent

Satellite images of the Gorgon Project sediment plume, of a quality suitable for deriving plume extent, were available for 411 of the 538 dredging days (i.e. 76% of days). Poor quality images (e.g., due to cloud cover or the sensor not capturing the study region) during the remaining 127 days (24% of days) were omitted from the analysis. Therefore, the number of sediment plume exposure days reported here is conservative and likely underestimate the true number of days sites spent under the sediment plume. Hotspot analysis of satellite imagery revealed that the sediment plume was most commonly detected around the dredge channel and sediment spoil dumping sites (Figure 1). Cumulative sediment plume exposure declined away from these sites, with the plume typically dispersing to the south of the dredge and spoil sites in response to prevailing wind and current patterns [30].

### Impact of dredging on coral disease prevalence

**Figure 2:**
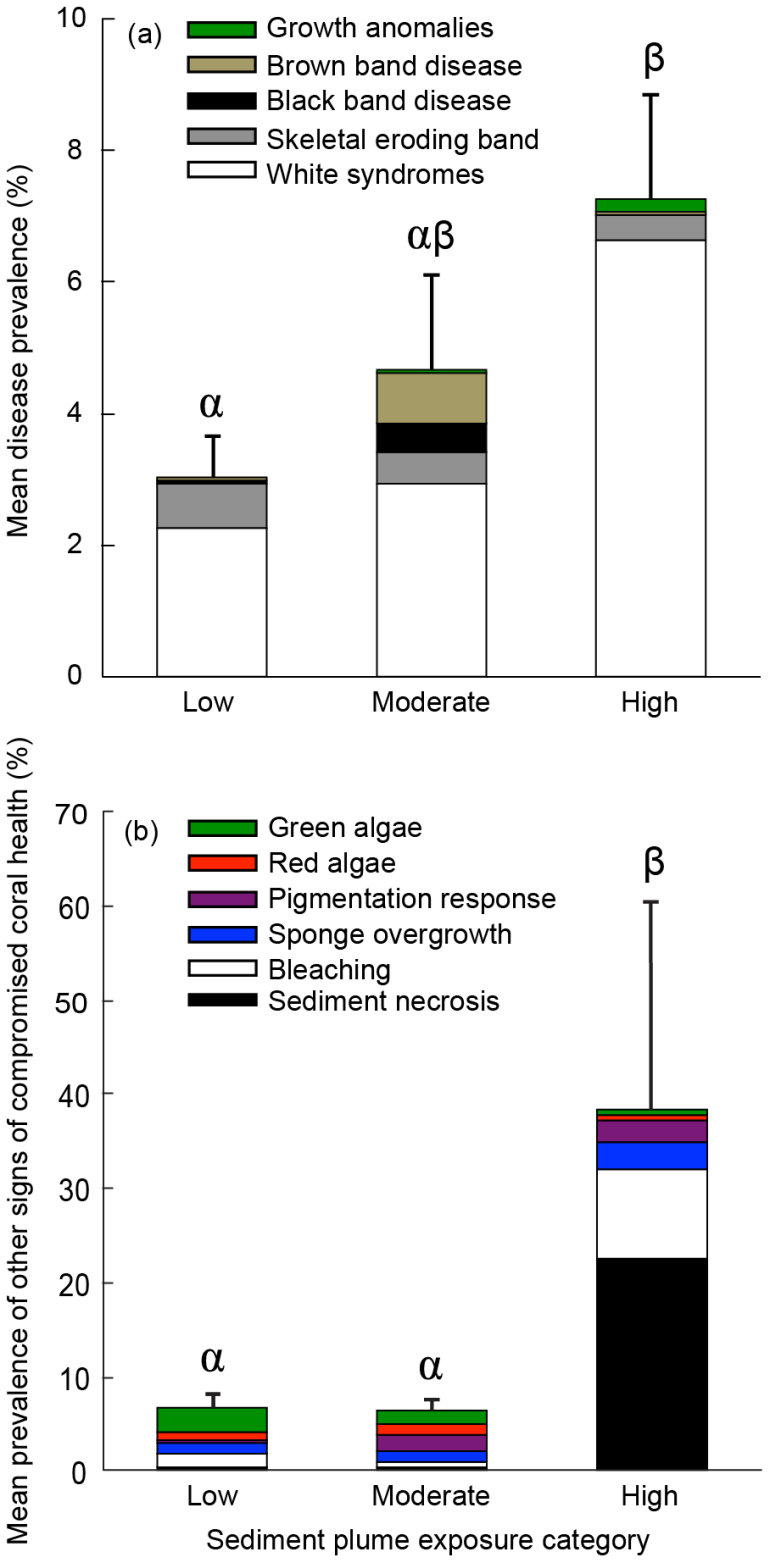
Mean prevalence of (a) coral disease and (b) other signs of compromised coral health at sites within three sediment plume exposure categories: low (0 to 9 plume exposure days; n = 18 transects), moderate (40 to 78 plume exposure days; n = 9 transects), and high (296 to 347 plume exposure days; n = 6 transects). Stacked bars indicate disease or other compromised coral health indicator prevalence by category and error bars indicate standard error among transects for total prevalence of disease or other compromised coral health indicators. Letters indicate post-hoc groupings (Tukey's HSD, *p<0.05*) between sediment plume exposure categories.

A significant, positive correlation was found between overall coral disease prevalence and sediment plume exposure days (Figure S1a in File S1, Pearson's *r*_9_ = 0.49, *p*<0.05). Mean disease prevalence (± SE) at high-exposure sites (7.26±1.56%) was greater than 2-fold higher than at low-exposure sites (3.1±0.6%) and 1.5-fold higher than at moderate-exposure sites (4.7±1.5%) (Figure 2a, Table S1 in File S1, F_2,8_ = 9.1, *p*<0.002). When results from all sites were combined, WS (69%) and skeletal eroding band (17%) dominated the disease cases observed. At the high-exposure sites, elevated disease prevalence was largely the result of high WS levels, which were 2.5-fold greater than at low- and moderate-exposure sites (Figure 2a, Table S1 in File S1, F_2,8_ = 17.5, *p<0.001*). In contrast, the highest prevalence of brown band disease was recorded at moderate-exposure sites, where it was more than 9 times greater than at high- or low-exposure sites (F_2,8_ = 0.9, *p<0.001*). The prevalence of black band disease, growth anomalies and skeletal eroding band did not differ significantly between exposure categories (Figure 2a, Table S1 in File S1, all *p>0.05*).

### Impact of dredging on other signs of compromised coral health

There was a significant, positive correlation between the prevalence of other compromised health indicators and sediment plume exposure days (Figure S1b in File S1, Pearson's *r_9_ = 0.79, p<0.001*). Mean prevalence of these indicators was more than 6-fold greater at high-exposure sites (47.9%±11.2) than at low- (8.0±1.4%) or moderate-exposure sites (7.9±0.9%) (Figure 2b, Table S1 in File S1, F_2,8_ = 50.8, *p<0.001*). Sediment-associated tissue necrosis was 57 times more prevalent at high-exposure sites compared to low- and moderate-exposure sites (Figure 2b, F_2,8_ = 154.9, *p<0.001*). Bleaching, sponge overgrowth and pigmentation responses were also significantly greater at high-exposure sites relative to low- or moderate-exposure sites (Figure 2b, Table S1 in File S1, all *p<0.001*). The prevalence of red and green algae did not differ significantly between exposure categories (all *p>0.05*).

### Influence of coral community composition and morphology on disease and other signs of compromised health

There was no significant difference in coral community composition between sediment plume exposure categories, indicating that reefs within the three groupings were comparable in regards to coral structure (Figure S2 in File S1, pseudo-F = 1.38, *p>0.1*). However, coral community composition did vary significantly among sites within exposure categories (Figure S2 in File S1, pseudo-F = 7.54, *p<0.001*).

Disease levels did not differ significantly among growth forms (i.e., massive, plating and branching colonies) at high or low exposure sites (Figure S3a in File S1, all *p>0.05*). However, massive corals at moderate-exposure sites sustained significantly less disease than branching and plating colonies (Figure S3a in File S1, all *p>0.05*). The prevalence of other compromised coral health indicators did not differ between growth forms within any sediment plume exposure category (Figure S3b in File S1, all *p>0.05*).

### Environmental drivers of disease and compromised health

ANOVA and DISTLM (visualized through dbRDA) both identified sediment plume exposure level as the most significant environmental driver of elevated levels of disease and other indicators of compromised health. Among all environmental parameters assessed (see Table 1), sediment plume exposure days was the only metric that differed significantly among exposure categories (F_2,11_ = 285.7, *p<0.001*). Furthermore, the abundance of disease predicted by satellite-derived temperature-based stress metrics did not differ significantly among sediment plume exposure groups (Table 1, *p>0.05*).

**Figure 3:**
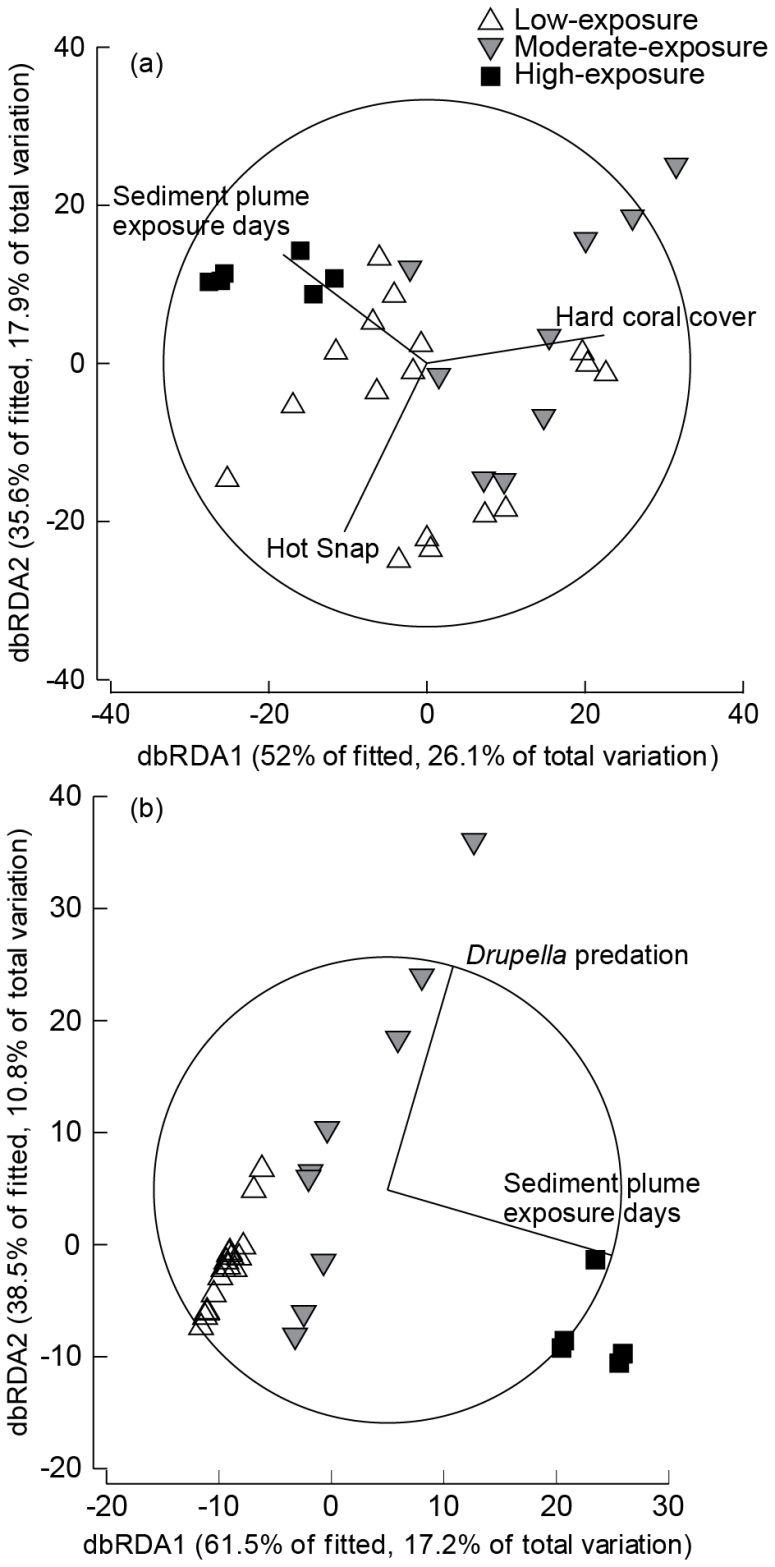
Distance-based redundancy analysis (dbRDA) ordination plots illustrating the relationship between environmental predictors that best explain the variation of (a) coral disease and (b) other compromised coral health indicators among sites. The dbRDA was constrained by the best-fit explanatory variables from a multivariate multiple regression analysis (DISTLM) and vectors overlays are shown for predictor variables explaining a significant proportion of the variation in the prevalence of (a) coral disease and (b) other compromised coral health indicators.

The dbRDA diagram depicting coral disease composition (based on the simplest best fit DISTLM, *AICc = 215.12, R^2^ = 0.47*), showed a cluster of high-exposure sites away from moderate- and low-exposure sites (Figure 3a). The greatest amount of variation in the disease prevalence data was explained by sediment plume exposure days (*pseudo-F_4,30_ = 3.97*, *p<0.05*) and total hard coral cover (*pseudo-F_4,30_ = 4.28, p<0.05*) (Figure 3a). The temperature metric Hot Snap (*pseudo-F_4,30_ = 3.31, p<0.05*) explained a lesser, but still significant amount of variation in the disease dataset (Figure 3a). The overlay vector for sediment plume exposure days corresponded to the axis separating high-exposure sites from low- and moderate-exposure sites, while the vectors for hard coral cover and Hot Snap largely corresponded to the axis separating sites within low- and moderate-exposure categories (Figure 3a).

The dbRDA diagram depicting the composition of other compromised health indicators (based on the simplest best fit DISTLM, *AICc = 215.57, R^2^ = 0.28*) also showed a clear separation between tightly clustered high-exposure transects and the more dispersed low- and moderate-exposure transects (Figure 3b). Sediment plume exposure days (*pseudo-F_4,30_ = 5.99, p<0.005*) and *Drupella* (*pseudo-F_4,30_ = 3.65, p<0.05*) explained the greatest amount of variation in the datacloud. The overlay vector for plume exposure days corresponded to the axis separating high-exposure sites from low- and moderate-exposure sites, while the vector for *Drupella* largely corresponded to the axis separating sites within low and moderate-exposure categories (Figure 3b).

## Discussion

This study provides the first empirical evidence linking turbidity and sedimentation with elevated levels of coral disease and other indicators of compromised coral health *in situ*. We found two-fold higher disease prevalence, largely driven by increases in WS, and six-fold higher levels of other compromised health indicators at high sediment plume exposure sites. Since these *in situ* health assessments were conducted more than 18 months after commencement of dredging, it is likely that the most susceptible corals experienced complete mortality prior to surveys being undertaken. Therefore, these prevalence figures likely underestimate the true impact of dredging-associated sedimentation and turbidity on coral health.

While previous studies have suggested a myriad of environmental stressors as potential drivers of coral disease [21], [22], [38], the current study highlights a direct link to sedimentation and turbidity. On Australia's east coast, the UNESCO World Heritage Committee recently cited increasing coastal development and catchment runoff as serious threats to the “outstanding universal value” of Australia's Great Barrier Reef World Heritage Area [39], and on Australia's west coast, an estimated 200 million cubic meters of sediment will be dredged and dumped in projects currently passing through Western Australia's approvals and/or regulatory system alone [40]. Clearly, these findings will have direct implications for coastal managers charged with balancing economic development with the imperative to maintain healthy coral reefs. As the rate of coastal development near coral reef ecosystems continues to escalate in many parts of the world, a comprehensive understanding of the impacts of sediment and turbidity on coral health will become increasingly important.

### Impact of dredging on coral disease prevalence

Elevated disease levels at high sediment plume exposure sites were primarily the result of the more than 2.5-fold higher prevalence of WS, an important group of diseases that have affected reefs throughout the Indo-Pacific and which are characterized by a distinct band of sloughing coral tissue revealing underlying coral skeleton [32], [41], [42]. WS prevalence is often correlated with environmental stress, and strong correlations between warm temperature anomalies and elevated WS levels have shown that thermal stress is an important driver of some types of WS [34], [35]. However, we found no differences in multiple thermal stress metrics or predicted disease abundance among the three sediment plume exposure groups and while Hot Snap and total hard coral cover helped to explain the distribution of disease among sites, this metric largely correlated with differences among low and moderate-exposure sites. Both univariate analysis and multivariate modeling identified sediment plume exposure as the main driver of elevated WS levels in the current study, providing further evidence for the role of environmental stress, specifically increased sedimentation and turbidity, in WS pathogenesis.

The greater prevalence of brown band disease at moderate-exposure sites compared to high- and low-exposure sites may reflect differences in ciliate proliferation rates under specific turbidity/light levels. Brown band disease is characterized by a dense, brown mat of ciliates packed with *Symbiodinium* derived from consumed coral tissue [32], [43]. Since *Symbiodinium* cells residing within ciliates are photosynthetically competent during brown band disease progression, it has been proposed that brown band disease-associated ciliates could derive nutrition from photosynthates produced by ingested *Symbiodinium*, while also benefiting from an additional oxygen source within the densely populated and presumably oxygen-limited brown band mat [32], [43]. At highly turbid, high sediment plume exposure sites, *Symbiodinium* photosynthesis would be hindered, potentially removing the advantage of brown band ciliates over their presumably immune-compromised coral hosts. However, medium-exposure sites could provide the right balance of compromised host immunity and sufficient light availability to facilitate infection and proliferation of brown band ciliates. Further studies specifically investigating the influence of reduced light levels on the partitioning of photosynthates between *Symbiodinium* and ciliates are required to test this hypothesis.

Total mean disease levels at low-exposure sites (3.1±0.6%) were similar to levels reported from nearby Ningaloo Marine Park [44], indicating that these low-exposure sites provide a good approximation of background disease levels in the region. Prevalence levels of black band disease, skeletal eroding band disease and growth anomalies did not differ significantly among sediment plume exposure groups and all were consistent with levels reported from Ningaloo Reef [44].

### Impact of dredging on other compromised coral health indicators

The greater prevalence of other indicators of compromised coral health at high sediment plume exposure sites was largely the result of elevated levels of sediment-associated necrosis and bleaching, which were 57-fold and 9-fold higher, respectively. Increased turbidity reduces the amount of light available for photosynthesis, while sediment deposition further shades corals and taxes energy budgets through the need to allocate energy to sediment removal. Although corals are able to actively remove sediment particles through ciliary and tentacular movement, combined with polyp distension and mucus production [45]–[47], these mechanisms can become overwhelmed during periods of intense and/or chronic sediment deposition. When sediment stress is chronic, even low-levels can dramatically alter coral energy budgets by reducing *Symbiodinium* densities (i.e., bleaching) and by decreasing the photochemical efficiencies (F_v_/F_m_) of the *Symbiodinium* that remain [45]–[47]. If resulting energy deficits are not relieved through either metabolic depression or heterotrophic feeding, bleaching can lead to mortality of the affected coral tissue (i.e., sediment necrosis).

While bleaching and sediment necrosis observed in this study were mostly confined to depressions on the coral surface, these patches of partial mortality could expose the coral to further mortality or subsequent infection by disease, even after the completion of dredging operations. In laboratory sedimentation experiments, Flores et al. [48] reported that corals with only 10% partial mortality at the end of a period of sediment exposure subsequently suffered complete mortality during a 4-week “recovery” period. Although bleaching and sediment necrosis are known sources of coral mortality during periods of prolonged sediment exposure [49], [50], future studies should investigate the potential of sediment-induced lesions to develop into coral disease, which could progress even after the sediment stress is removed.

Elevated prevalence of pigmentation responses (PR) at high exposure sites provides further evidence of sediment plume-induced coral stress. PR has been observed in corals subjected to a suite of stressors and has been proposed to be a general immune response to a variety of physical and biological challenges, including competitors and pathogens [32], [51], [52]. Tissues associated with PR possess high levels of melanin, an important component of invertebrate innate immunity that can act as a defensive barrier against foreign bodies [52]. Elevated PR levels at high exposure sites may represent an inflammatory-like response by the coral to either sediment particles clogging tentacles and polyp surfaces or to invading pathogens.

### Effect of coral growth form on sediment plume-induced disease and other compromised health indicators

The prevalence of disease and other compromised coral health indicators did not vary between coral growth forms (i.e., massive, plating and branching) at high sediment plume exposure sites. The influence of coral morphology (i.e., growth from) on sediment clearing rates has been well investigated [45], [53], [54], with branching corals generally considered to be more effective at passive sediment removal, while massive and plating forms retain more sediment due to their shapes, which inhibit passive rejection and removal [55]. However, sediment rejection rates and sediment tolerance are not directly related [54]. For example, Stafford-Smith [54] reported some plating species (e.g. *Montipora aequituberculata*) with high sediment tolerance despite poor sediment rejection capacity, whereas some massive species (e.g. *Favia stelligera* and *Leptoria phrygia*) are efficient sediment rejecters but exhibit low sediment tolerance. While previous investigations have focused on only a few coral species in relatively artificial conditions, this is the first field study to investigate the relationship between growth form, sedimentation, turbidity and coral health among all hard coral species present on a natural reef. Although this study indicates that growth form is not a strong predictor of corals' susceptibility to disease and other compromised health indicators associated with increased sedimentation and turbidity, some caution is required in this interpretation, as the most susceptible corals may have died before these surveys were undertaken.

#### Conclusions

This study provides empirical, field-based evidence linking sedimentation and turbidity to elevated levels of coral disease and other compromised coral health indicators on reefs. While poor water quality has been suggested as a driver of coral disease [13], [56], [57], little ecological evidence exists linking specific water quality parameters and coral disease data. WS responded strongly to elevated sedimentation and turbidity demonstrating a clear link between water quality and coral disease, though the mechanisms underlying this response remain unclear. Future studies that investigate the response of the coral host (e.g., immune function and energy reserves) and potential pathogens (e.g., shifts in bacterial and viral communities on corals and in surrounding seawater) to elevated sedimentation and turbidity are required. As coastal development intensifies in many parts of the world, the health of coral reefs will depend upon a thorough understanding of the impacts of water quality changes on coral reef health.

## Supporting Information

File S1 **This file contains supplementary Methods S1, Discussion, Table S1, and Figure S1–Figure S3.** Methods S1, Coral genera classified by growth form. Table S1, Mean prevalence of individual coral diseases and other health indicators. Figure S1, Correlations between sediment plume exposure days and the prevalence of disease and other compromised coral health indicators. Figure S2, Non-metric multidimensional scaling (nMDS) plot of coral assemblages. Figure S3, Prevalence of disease and other compromised health by growth form.
(DOCX)
