# Correction: Sediment and Turbidity Associated with Offshore Dredging Increase Coral Disease Prevalence on Nearby Reefs

**DOI:** 10.1371/journal.pone.0165541

**Published:** 2016-11-01

**Authors:** F. Joseph Pollock, Joleah B. Lamb, Stuart N. Field, Scott F. Heron, Britta Schaffelke, George Shedrawi, David G. Bourne, Bette L. Willis

Following a re-evaluation of the hierarchical analyses of variance (nested ANOVA) performed to test for differences in coral health and disease among dredge-exposure categories, it was discovered that sites were not treated as a random effect in the model when analyses were run using the software package Statistica. As a consequence, incorrect denominator MS's were used in calculations of F ratios. Following consultation with a number of expert statisticians, it was concluded that it is more appropriate to perform a generalized linear mixed model (GLMM) on response variables with binomial distributions in studies with random effects, such as counts of disease at random sites. The GLMM procedure simultaneously incorporates the random effects modelling of ANOVA and the appropriate modelling of categorical response variables that one gets from a regression. Therefore, the coupled use of a nested ANOVA and Pearson Product Moment Correlation (PPMC) has been replaced with the recommended GLMM. Additionally, in order to make the results readily interpretable to managers and policy makers, a Bayesian regression approach has been incorporated to infer probabilistic likelihoods of differences in coral health and disease amongst dredge-exposure categories. Many advocate the tandem use of both frequentist (GLMM) and Bayesian approaches due to their complementary merits. Use of these two approaches strengthens the study’s conclusions, and make the results more accessible to reef managers and stakeholders requiring knowledge of the impacts of dredging on coral health.

The revised statistical methodology is described in the revised Methods section and in the revised S1 File. The results of these revised statistical analyses are laid out in revised Table S1 (S1 File) and in the relevant sections of the revised Results section.

The final paragraph of the Methods section should read:

To analyze patterns of coral disease and other signs of compromised health among broad coral growth forms within each sediment plume exposure category, coral genera were assigned to one of three growth form categories: massive, plating or branching (see Methods S1 in S1 File). Differences in mean prevalence levels among the three sediment plume exposure groups were analyzed using generalized linear mixed-effects models (GLMMs) with a binomial (logit link) error distribution. Sediment exposure categories were treated as fixed effects, while site and transect were incorporated as random effects. The GLMM was developed using the glmer function of the lme4 package implemented in R and p-values were calculated with Wald tests for mixed models [2]. Pair-wise differences in the prevalence of disease and other compromised coral health indicators between sediment plume exposure groups were also analyzed using a Bayesian hierarchical linear mixed model with a binomial distribution using JAGS [3] implemented in R [2] with the package R2jags [4] (see Supplementary Methods in S1 File). Plume exposure days, coral predation by COTS and Drupella, and total hard coral cover were compared among plume exposure groups using one-way ANOVAs. Differences in mean prevalence of disease and compromised health indicators were compared among growth forms within each dredge exposure category using one-way ANOVAs. Similarly, all temperature-based measures of disease likelihood were compared using one-way ANOVAs. Prior to analyses, assumptions of normality (Shapiro-Wilks) and homogeneity of variance (Levene’s test of homogeneity) were tested. Post-hoc comparisons between groups were performed using Tukey’s HSD tests. All univariate statistical analyses were performed using the R statistical program [2].

The second through sixth paragraphs of the Results section should read:

## Impact of dredging on coral disease prevalence

Mean disease prevalence (± SE) at high-exposure sites (7.26±1.56%) was greater than 2-fold higher than at low-exposure sites (3.1±0.6%, GLMM: z = -1.74, p = 0.08) and 1.5-fold higher than at moderate-exposure sites (4.7±1.5%, GLMM: z = -1.12, p = 0.26) (Fig 2a, Table S1 in S1 File). Bayesian hierarchical modelling indicates that this corresponds to a 90% probability that overall coral disease prevalence is greater at the high- than the low-exposure sites, and an 83% probability that disease is greater at the high- than the moderate-exposure sites (Table S1 in S1 File). When results from all sites were combined, WS (69%) and skeletal eroding band (17%) dominated the disease cases observed. At the high-exposure sites, elevated disease prevalence was largely the result of high WS levels, which were 2.5-fold greater than at low- and moderate- exposure sites (Fig 2a, Table S1 in S1 File, High v. Low: z = -1.71, p = 0.09, Bayesian = 91% probability; High v. Moderate: z = -1.76, p = 0.08, Bayesian = 92% probability). In contrast, the highest prevalence of brown band disease was recorded at moderate-exposure sites, where it was nearly 9 times greater than at high- (GLMM: z = 1.14, p = 0.25, Bayesian modeling: 13% probability) or low-exposure sites (GLMM: z = 2.01, p = 0.04, Bayesian modeling: 95% probability). The prevalence of black band disease and skeletal eroding band did not differ significantly between exposure categories (Fig 2a, Table S1 in S1 File, all p>0.10, Bayesian modeling: generally, <90% probability).

**Fig 2:**
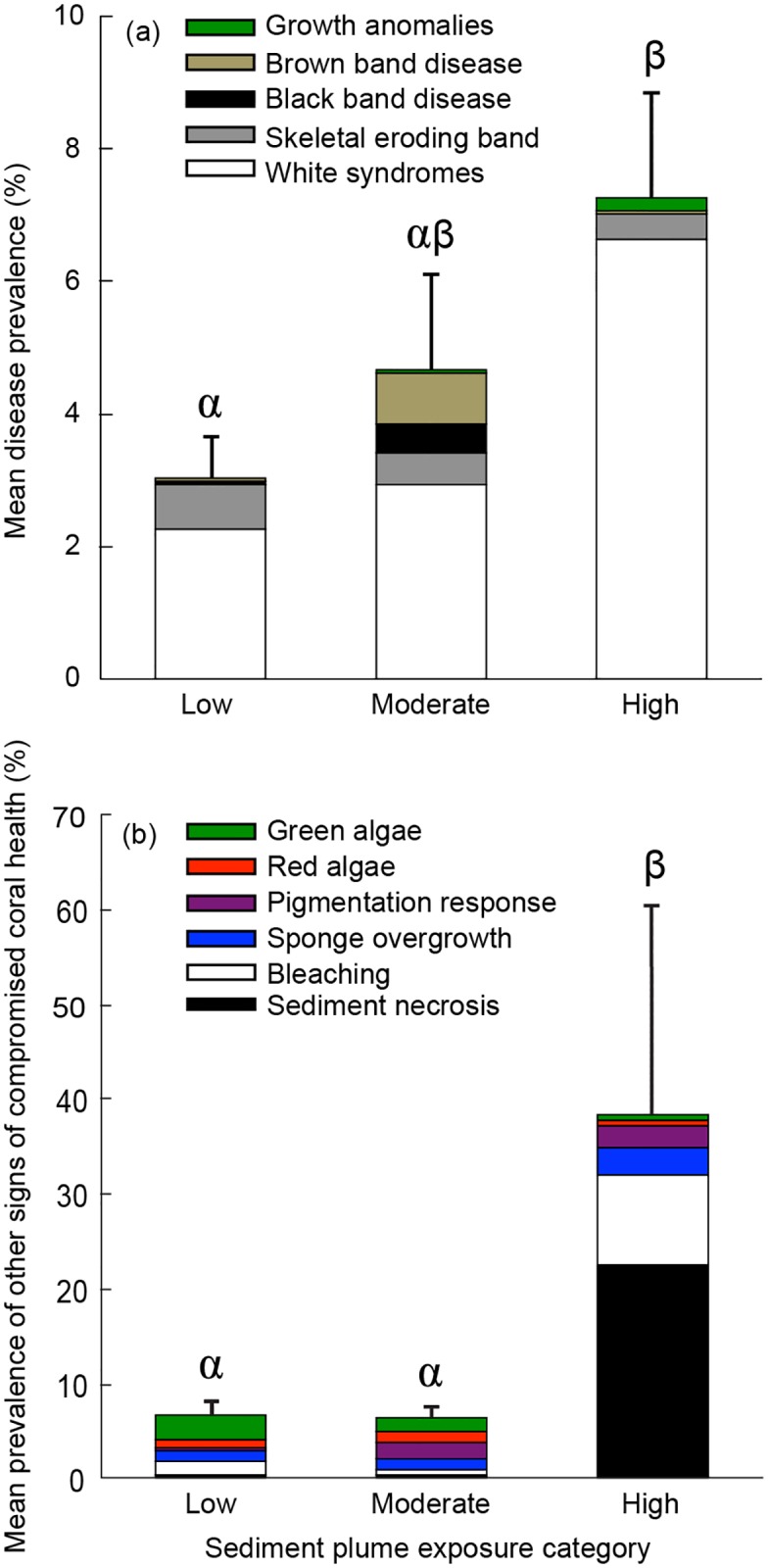
Mean prevalence of (a) coral disease and (b) other signs of compromised coral health at sites within three sediment plume exposure categories: low (0 to 9 plume exposure days; n = 18 transects), moderate (40 to 78 plume exposure days; n = 9 transects), and high (296 to 347 plume exposure days; n = 6 transects). Stacked bars indicate disease or other compromised coral health indicator prevalence by category and error bars indicate standard error among transects for total prevalence of disease or other compromised coral health indicators. Letters indicate ≥ 90% probability of differences (by Bayesian hierarchical modeling) between sediment plume exposure categories.

## Impact of dredging on other signs of compromised coral health

Mean prevalence of other compromised coral health indicators was more than 6- fold greater at high-exposure sites (38.3%±23.4) than at low- (6.6±1.7%, GLMM: z = -3.4, p = 0.00, Bayesian modeling: 98% probability) or moderate-exposure sites (6.4±1.1%, GLMM: z = -2.72, p = 0.01, Bayesian modeling: 96% probability) (Fig 2b, Table S1 in S1 File). Sediment-associated tissue necrosis was 57 times more prevalent at high-exposure sites compared to low- and moderate-exposure sites (Fig 2b, High v. Low: z = -3.65, p = 0.00, Bayesian = 99% probability; High v. Moderate: z = -3.91, p = 0.00, Bayesian = 100% probability). Bleaching and pigmentation responses were also significantly greater at high- exposure sites relative to low-exposure sites (Fig 2b, Table S1 in S1 File, all p<0.001, Bayesian modeling: ≥ 99% probability). The prevalence of red and green algae did not differ significantly between exposure categories (all p>0.10, Bayesian modeling: generally, ≤ 40% probability).

## Influence of coral community composition and morphology on disease and other signs of compromised health

There was no significant difference in coral community composition between sediment plume exposure categories, indicating that reefs within the three groupings were comparable in regards to coral structure (Figure S1 in S1 File, pseudo-F = 1.38, p>0.1). However, coral community composition did vary significantly among sites within exposure categories (Figure S1 in S1 File, pseudo-F = 7.54, p<0.001).

Disease levels did not differ significantly among growth forms (i.e., massive, plating and branching colonies) at high or low exposure sites (Figure S2a in S1 File, all p>0.05). However, massive corals at moderate-exposure sites sustained significantly less disease than branching and plating colonies (Figure S2a in S1 File, all p<0.05). The prevalence of other compromised coral health indicators did not differ between growth forms within any sediment plume exposure category (Figure S2b in S1 File, all p>0.05).

## Environmental drivers of disease and compromised health

ANOVA and DISTLM (visualized through dbRDA) both identified sediment plume exposure level as the most significant environmental driver of elevated levels of disease and other indicators of compromised health. Among all environmental parameters assessed (see Table 1), sediment plume exposure days was the only metric that differed significantly among exposure categories (F2,8 = 285.7, p<0.001). Furthermore, the abundance of disease predicted by satellite-derived temperature-based stress metrics did not differ significantly among sediment plume exposure groups (Table 1, p>0.05).

The legend for Fig 2 is updated. Please read the corrected Fig 2 legend here:

The final sentence of the fifth paragraph of the Discussion section should read: Prevalence levels of black band disease and skeletal eroding band disease did not differ significantly among sediment plume exposure groups and both were consistent with levels reported from Ningaloo Reef.

Additional citations are found in the Reference section.

S1 File has been updated. Please see the corrected Supporting Information file below.

## Supporting Information

S1 File This file contains supplementary Methods S1, Table S1, and Figure S1–Figure S2. Methods S1, Coral genera classified by growth form and Bayesian model parameters. Table S1, Mean prevalence of individual coral diseases and other health indicators. Figure S1, Non-metric multidimensional scaling (nMDS) plot of coral assemblages. Figure S2, Prevalence of disease and other compromised health by growth form.
(DOCX)

## References

[1] Pollock FJ, Lamb JB, Field SN, Heron SF, Schaffelke B, Shedrawi G, et al (2014) Sediment and Turbidity Associated with Offshore Dredging Increase Coral Disease Prevalence on Nearby Reefs. PLoS ONE 9(7): e102498 doi: 10.1371/journal.pone.0102498 25029525 10.1371/journal.pone.0102498 PMC4100925

[2] R Development Core Team. (2015) R: A language and environment for statistical computing. Vienna, Austria: R Foundation for Statistical Computing.

[3] Plummer M. (2003) JAGS: A program for analysis of Bayesian graphical models using Gibbs sampling Proceedings of the 3rd International Workshop on Distributed Statistical Computing. Vienna, Austria: Technische Universit at Wien.

[4] Su Y-S, Yajima M. (2012) R2jags: A Package for Running jags from R. R package version 005–07, http://CRANR-project.org/package=R2jags.

