# Supplementary material for: Sediment and Turbidity Associated with Offshore Dredging Increase Coral Disease Prevalence on Nearby Reefs
Source: PLoS One. 2014 Jul 16;9(7):e102498. doi: 10.1371/journal.pone.0102498 (PMC4100925; doi:10.1371/journal.pone.0102498)
Supplement: File S1 — This file contains supplementary Methods S1, Discussion, Table S1, and Figure S1–Figure S3. Methods S1, Coral genera classified by growth form. Table S1, Mean prevalence of individual coral diseases and other health indicators. Figure S1, Correlations between sediment plume exposure days and the prevalence of disease and other compromised coral health indicators. Figure S2, Non-metric multidimensional scaling (nMDS) plot of coral assemblages. Figure S3, Prevalence of disease and other compromised health by growth form. (DOCX) [file pone.0102498.s001.docx]

**Supplementary Material**

**Sediment and turbidity associated with offshore dredging increase coral disease prevalence on nearby reefs**

F. Joseph Pollock, Joleah B. Lamb, Stuart N. Field, Scott F. Heron, Britta Schaffelke, George Shedrawi, David G. Bourne, Bette L. Willis

**Supplementary Section Contents**  **Page**

**1** Coral genera classified by growth form **(Methods)**……………………………………. 2

**2** Impact of dredging on other compromised coral health indicators (**Discussion**)….…..... 3-4

**3** Supplementary references………………………………………………………………. 5

**4** Mean prevalence of individual coral diseases and other health indicators **(Table S1)** ... 6

**5** Correlations between sediment plume exposure days and the prevalence of disease and other compromised coral health indicators **(Figure S1)**…….……………………. 7

**6** Non-metric multidimensional scaling (nMDS) plot of coral assemblages **(Figure S2)**.. 8

**7**  Prevalence of disease and other compromised health by growth form **(Figure S3)……** 9

**Growth form categories and assigned coral genera (Methods):**

**(1) Massive** - *Acanthastrea*, *Alveopora*, *Astreopora*, *Cyphastrea*, *Diploastrea*, *Favia*, *Favites*, *Fungiidae*, *Goniastrea*, *Goniopora*, *Leptastrea*, *Leptoria*, *Leptoseris*, *Lobophyllia*, *Montastrea*, *Moseleya*, *Oulophyllia*, *Platygyra*, massive *Porites* and *Symphyllia;*

**(2) Plating** - tabular *Acropora*, *Echinophyllia*, plating *Echinopora, Galaxea*, *Merulina*, plating *Montipora*, *Mycedium*, *Oxypora*, *Pachyseris*, *Pectinia*, *Podabacia* and *Turbinaria*;

**(3) Branching** - bushy *Acropora*, digitate *Acropora*, staghorn *Acropora*, *Anacropora*, *Australogyra*, branching *Echinopora*, *Hydnophora*, *Isopora*, branching *Montipora*, *Palauastrea*, *Paraclavarina*, *Pavona*, branching *Pocillopora,* branching *Porites*, *Psammocora*, *Seriatopora* and *Stylophora*;

**Impact of dredging on other compromised coral health indicators (Discussion)**

Increased turbidity reduces the amount of light available for photosynthesis, while sediment deposition further shades corals and taxes energy budgets through the need to allocate energy to sediment removal. Although corals are able to actively remove sediment particles through ciliary and tentacular movement, combined with polyp distension and mucus production [1–3], these mechanisms can become overwhelmed during periods of intense and/or chronic sediment deposition. When sediment stress is chronic, even low-levels can dramatically alter coral energy budgets by reducing *Symbiodinium* densities (i.e., bleaching) and by decreasing the photochemical efficiencies (F_v_/F_m_) of the *Symbiodinium* that remain [1–3]. If resulting energy deficits are not relieved through either metabolic depression or heterotrophic feeding, bleaching can lead to mortality of the affected coral tissue (i.e., sediment necrosis).

While bleaching and sediment necrosis observed in this study were mostly confined to depressions on the coral surface, these patches of partial mortality could expose the coral to further mortality or subsequent infection by disease, even after the completion of dredging operations. In laboratory sedimentation experiments, Flores et al. [4] reported that corals with only 10% partial mortality at the end of a period of sediment exposure subsequently suffered complete mortality during a 4-week “recovery” period. Although bleaching and sediment necrosis are known sources of coral mortality during periods of prolonged sediment exposure [5,6], future studies should investigate the potential of sediment-induced lesions to develop into coral disease, which could progress even after the sediment stress is removed.

Elevated prevalence of pigmentation responses (PR) at high exposure sites provides further evidence of sediment plume-induced coral stress. PR has been observed in corals subjected to a suite of stressors and has been proposed to be a general immune response to a variety of physical and biological challenges, including to competitors and pathogens [7–10]. Tissues associated with PR possess high levels of melanin, an important component of invertebrate innate immunity that can act as a defensive barrier against foreign bodies [10]. Elevated PR levels at high exposure sites may represent an inflammatory-like response by the coral to either sediment particles clogging tentacles and polyp surfaces or to invading pathogens.

**Supplementary References**

1. Hubbard JAEB, Pocock YP (1972) Sediment rejection by recent scleractinian corals: a key to palaeo-environmental reconstruction. Geol Rundsch 61: 598–626. doi:10.1007/BF01896337.

2. Rogers C (1990) Responses of coral reefs and reef organisms to sedimentation. Mar Ecol Prog Ser 62: 185–202. doi:10.3354/meps062185.

3. Philipp E, Fabricius K (2003) Photophysiological stress in scleractinian corals in response to short-term sedimentation. J Exp Mar Biol Ecol 287: 57–78. doi:10.1016/S0022-0981(02)00495-1.

4. Flores F, Hoogenboom MO, Smith LD, Cooper TF, Abrego D, et al. (2012) Chronic exposure of corals to fine sediments: lethal and sub-lethal impacts. PloS One 7: e37795. doi:10.1371/journal.pone.0037795.

5. Glynn PW (1996) Coral reef bleaching: facts, hypotheses and implications. Glob Change Biol 2: 495–509. doi:10.1111/j.1365-2486.1996.tb00063.x.

6. Brown BE (1997) Coral bleaching: causes and consequences. Coral Reefs 16: 129–138. doi:10.1007/s003380050249.

7. Willis BE, Page CA, Dinsdale EA (2004) Coral Disease on the Great Barrier Reef. Coral Health and Disease. Berlin: Springer-Verlag Publishing. pp. 69–104.

8. Bongiorni L, Rinkevich B (2005) The pink-blue spot syndrome in Acropora eurystoma (Eilat, Red Sea): A possible marker of stress? Zoology 108: 247–256. doi:10.1016/j.zool.2005.05.002.

9. Ravindran J, Raghukumar C (2006) Pink-line syndrome, a physiological crisis in the scleractinian coral Porites lutea. Mar Biol 149: 347–356. doi:10.1007/s00227-005-0192-1.

10. Palmer CV, Mydlarz LD, Willis BL (2008) Evidence of an inflammatory-like response in non-normally pigmented tissues of two scleractinian corals. Proc R Soc B Biol Sci 275: 2687–2693. doi:10.1098/rspb.2008.0335.

**Table S1.** Mean prevalence of coral disease and compromised coral health indicators at sites within three sediment plume exposure categories determined by MODIS satellite imagery: low (0 to 9 plume exposure days), moderate (40 to 68 plume exposure days) and high (296 to 347 plume exposure days) and results of a 2-way nested analysis of variance (ANOVA). Mean prevalence calculated as the percentage of colonies with disease or compromised health as a percentage of the total number of corals per transect. Analyses performed on square root transformed data.

| **Factor** | **Sediment Plume Exposure Category** | | | | | | |  | |  |
| --- | --- | --- | --- | --- | --- | --- | --- | --- | --- | --- |
|  | **Low**  (n = 18 transects) |  | **Moderate** (n = 9 transects) |  | **High** (n = 6 transects) |  | **ANOVA** | | | |
|  | mean (SE) prevalence (%) |  | mean (SE) prevalence (%) |  | mean (SE) prevalence (%) |  | **Exposure** | | **Site (exposure)** | |
|  |  |  |  |  |  |  | ***F, p*** | | ***F, p*** | |
| Total disease | 3.1 (0.6) |  | 4.7 (1.5) |  | 7.3 (1.6) |  | 9.1, <0.002* | | 7.3, <0.001* | |
| White syndromes | 2.3 (0.5) |  | 2.9 (1.2) |  | 6.7 (1.2) |  | 17.5, <0.001* | | 10.4, <0.001* | |
| Brown band | 0.07 (0.04) |  | 0.8 (0.4) |  | 0.09 (0.09) |  | 0.9, <0.001* | | 5.7, <0.005* | |
| Black band | 0.03 (0.02) |  | 0.5 (0.4) |  | 0.0 (0.0) |  | 2.7, <0.09 | | 1.6, <0.17 | |
| Skeletal eroding band | 0.7 (0.2) |  | 0.5 (0.2) |  | 0.4 (0.3) |  | 1.4, <0.27 | | 2.3, <0.06 | |
| Growth anomalies | 0.0 (0.0) |  | 0.03 (0.03) |  | 0.2 (0.2) |  | 1.6, <0.22 | | 0.7, <0.66 | |
| Total compromised health | 8.0 (1.4) |  | 7.9 (0.9) |  | 47.9 (11.2) |  | 50.8, <0.001* | | 8.1, <0.001* | |
| Sediment necrosis | 0.5 (0.2) |  | 0.3 (0.2) |  | 22.7 (8.5) |  | 154.9, <0.001* | | 16.0, <0.001* | |
| Bleaching | 1.4 (0.4) |  | 0.7 (0.2) |  | 9.5 (3.8) |  | 20.1, <0.001* | | 3.0, <0.02* | |
| Sponge overgrowth | 1.0 (0.3) |  | 1.2 (0.4) |  | 2.8 (0.3) |  | 6.9, <0.005* | | 3.3, <0.01* | |
| Pigmentation response | 0.3 (0.1) |  | 1.6 (0.5) |  | 2.4 (0.3) |  | 24.8, <0.001* | | 2.5, <0.04* | |
| Red algal overgrowth | 0.9 (0.4) |  | 1.2 (0.4) |  | 0.4 (0.3) |  | 1.8, <0.19 | | 2.2, <0.07 | |
| Green algal overgrowth | 2.5 (1.1) |  | 1.5 (0.8) |  | 0.5 (0.2) |  | 2.3, <0.12 | | 4.2, <0.003* | |

* denotes a significant difference for α = 0.05

**
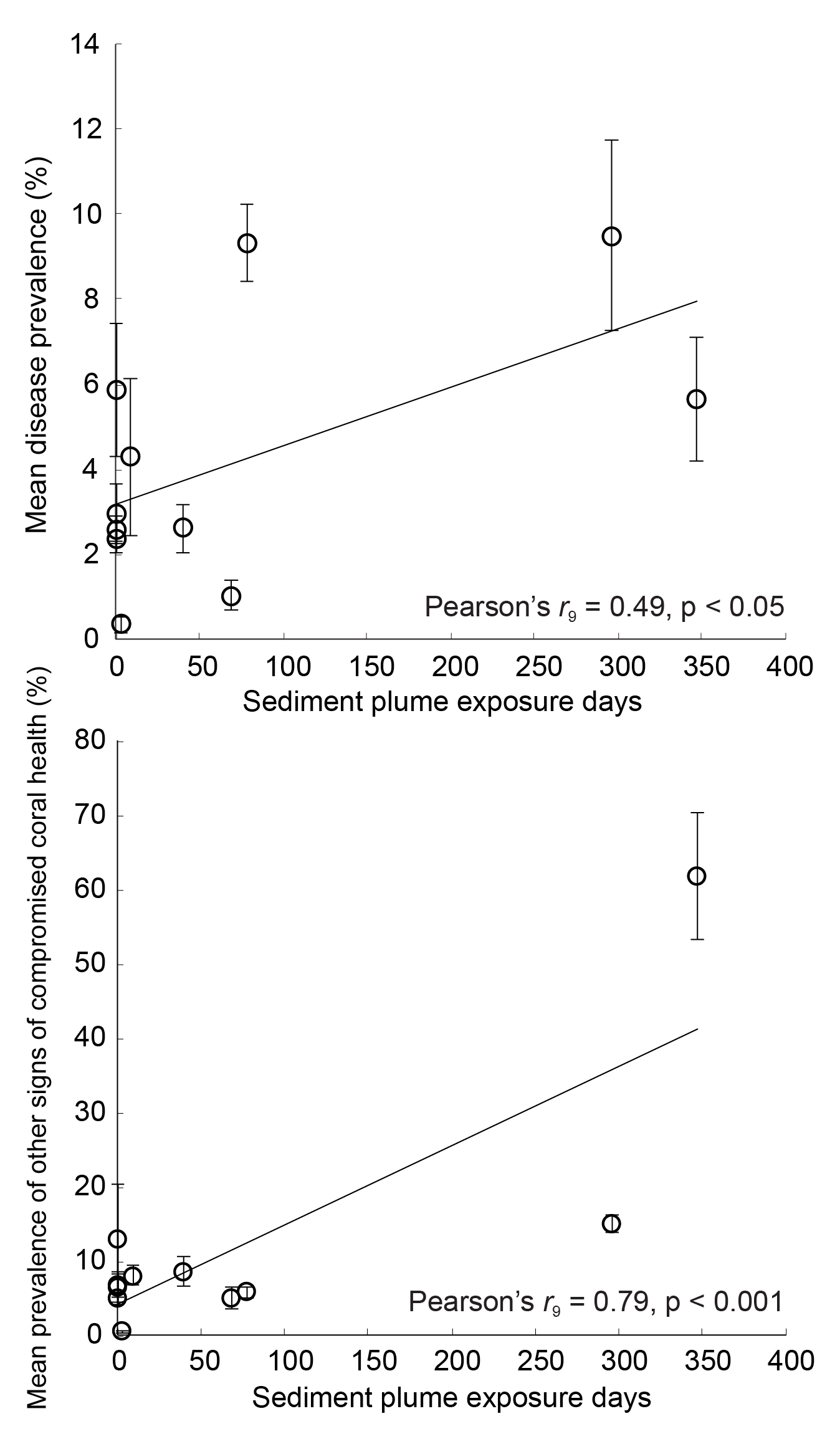
**

**Figure S1**. Correlations between sediment plume exposure days and the prevalence of (a) disease and (b) other signs of compromised coral health. Pearson's correlation coefficients (Pearson’s *r*) and p-values are shown.

**Figure S2.** Non-metric multidimensional scaling (nMDS) plot visualizing variation in the taxonomic composition (genus-level) of coral assemblages at transects within each sediment plume exposure category: low (0 to 9 plume exposure days; n = 18 transects; white triangles), moderate (40 to 78 plume exposure days; n = 9 transects; grey triangles), and high (296 to 347 plume exposure days; n = 6 transects; black squares).

**
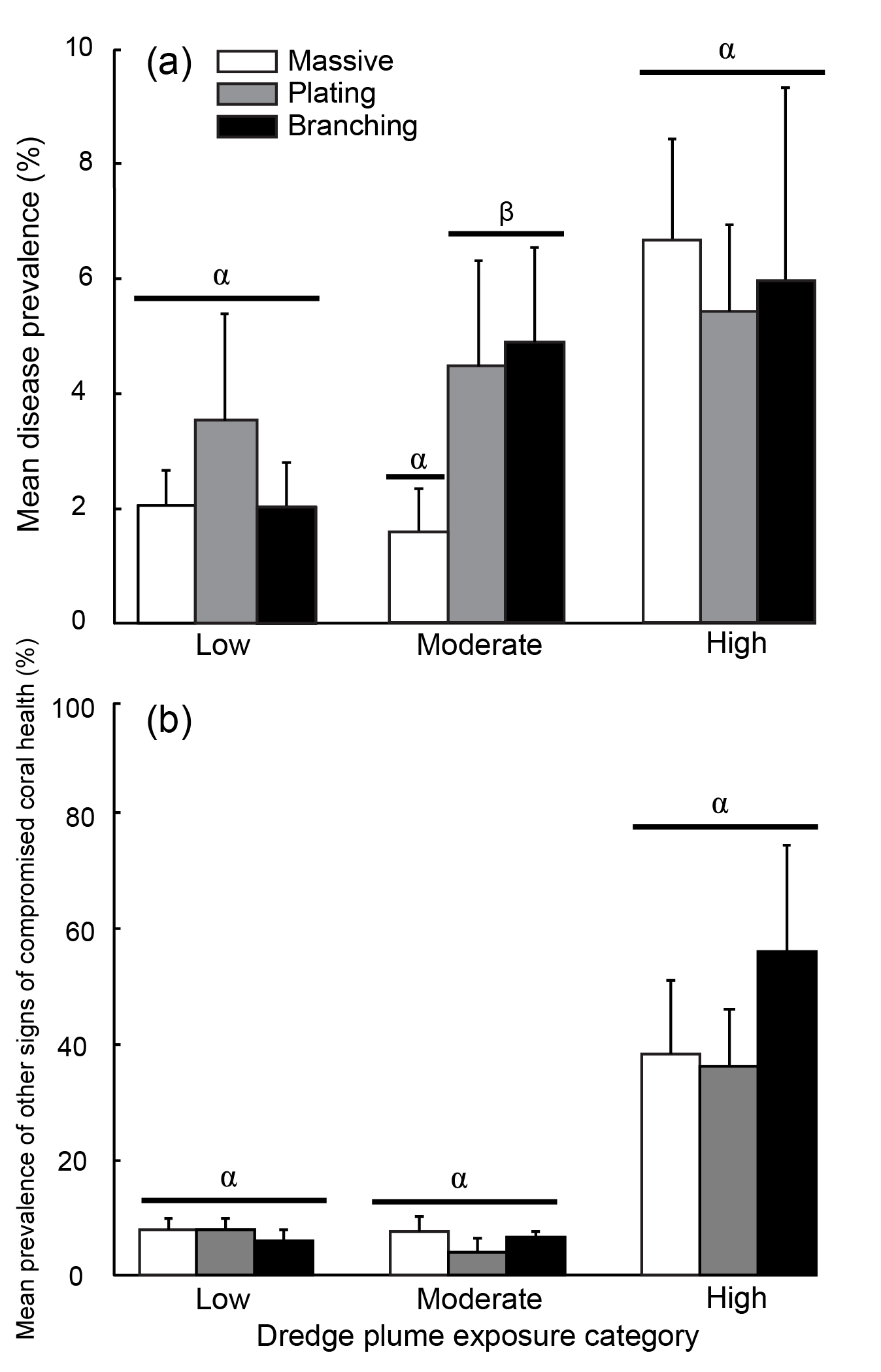
**

**Figure S3.** Mean prevalence of (a) coral disease and (b) other compromised coral health indicators at sites within three sediment plume exposure categories, low (0 to 9 plume exposure days; n = 15 transects; white bars), moderate (40 to 78 plume exposure days; n = 9 transects; grey bars) and high (296 to 347 plume exposure days; n = 6 transects; black bars), for three coral growth morphologies, massive (n = 1725 colonies), plating (n = 1768 colonies) and branching (n = 3351 colonies). Lettered bars indicate post-hoc groupings (*Tukey’s HSD, p < 0.05*) between morphologies within exposure categories.
