# Supplementary material for: Correction: Sediment and Turbidity Associated with Offshore Dredging Increase Coral Disease Prevalence on Nearby Reefs
Source: PLoS One. 2016 Nov 1;11(11):e0165541. doi: 10.1371/journal.pone.0165541 (PMC5089681; doi:10.1371/journal.pone.0165541)
Supplement: S1 File — Methods S1, Coral genera classified by growth form and Bayesian model parameters. Table S1, Mean prevalence of individual coral diseases and other health indicators. Figure S1, Non-metric multidimensional scaling (nMDS) plot of coral assemblages. Figure S2, Prevalence of disease and other compromised health by growth form. (DOCX) [file pone.0165541.s001.docx]

**Supplementary Material**

**Sediment and turbidity associated with offshore dredging increase coral disease prevalence on nearby reefs**

F. Joseph Pollock, Joleah B. Lamb, Stuart N. Field, Scott F. Heron, Britta Schaffelke, George Shedrawi, David G. Bourne, Bette L. Willis

**Supplementary Section Contents**  **Page**

**1** Coral genera classified by growth form **(Methods)**……………………………………. S2

**2**  Bayesian model parameters **(Methods)**……………………………………........….… S3

**3** Mean prevalence of individual coral diseases and other health indicators **(Table S1)** ... S4

**4** Non-metric multidimensional scaling (nMDS) plot of coral assemblages **(Figure S1)**.. S5

**5**  Prevalence of disease and other compromised health by growth form **(Figure S2)**…... S6

**6** Supplemental References **(References)**……………………………………………….. S7**Growth form categories and assigned coral genera (Supplementary Methods):**

**(1) Massive** - *Acanthastrea*, *Alveopora*, *Astreopora*, *Cyphastrea*, *Diploastrea*, *Favia*, *Favites*, *Fungiidae*, *Goniastrea*, *Goniopora*, *Leptastrea*, *Leptoria*, *Leptoseris*, *Lobophyllia*, *Montastrea*, *Moseleya*, *Oulophyllia*, *Platygyra*, massive *Porites* and *Symphyllia;*

**(2) Plating** - tabular *Acropora*, *Echinophyllia*, plating *Echinopora, Galaxea*, *Merulina*, plating *Montipora*, *Mycedium*, *Oxypora*, *Pachyseris*, *Pectinia*, *Podabacia* and *Turbinaria*;

**(3) Branching** - bushy *Acropora*, digitate *Acropora*, staghorn *Acropora*, *Anacropora*, *Australogyra*, branching *Echinopora*, *Hydnophora*, *Isopora*, branching *Montipora*, *Palauastrea*, *Paraclavarina*, *Pavona*, branching *Pocillopora,* branching *Porites*, *Psammocora*, *Seriatopora* and *Stylophora*;

**Bayesian model parameters (Supplementary Methods):**

Beta-binomial models equivalent to the glmer models described for GLMM analyses were also fit in a Bayesian framework to explore pair-wise differences in the prevalence of disease and other compromised coral health indicators between sediment plume exposure groups. Logistic models were used for black band disease, brown band disease and growth anomaly to account for zero inflation in these datasets.

| Logistic:  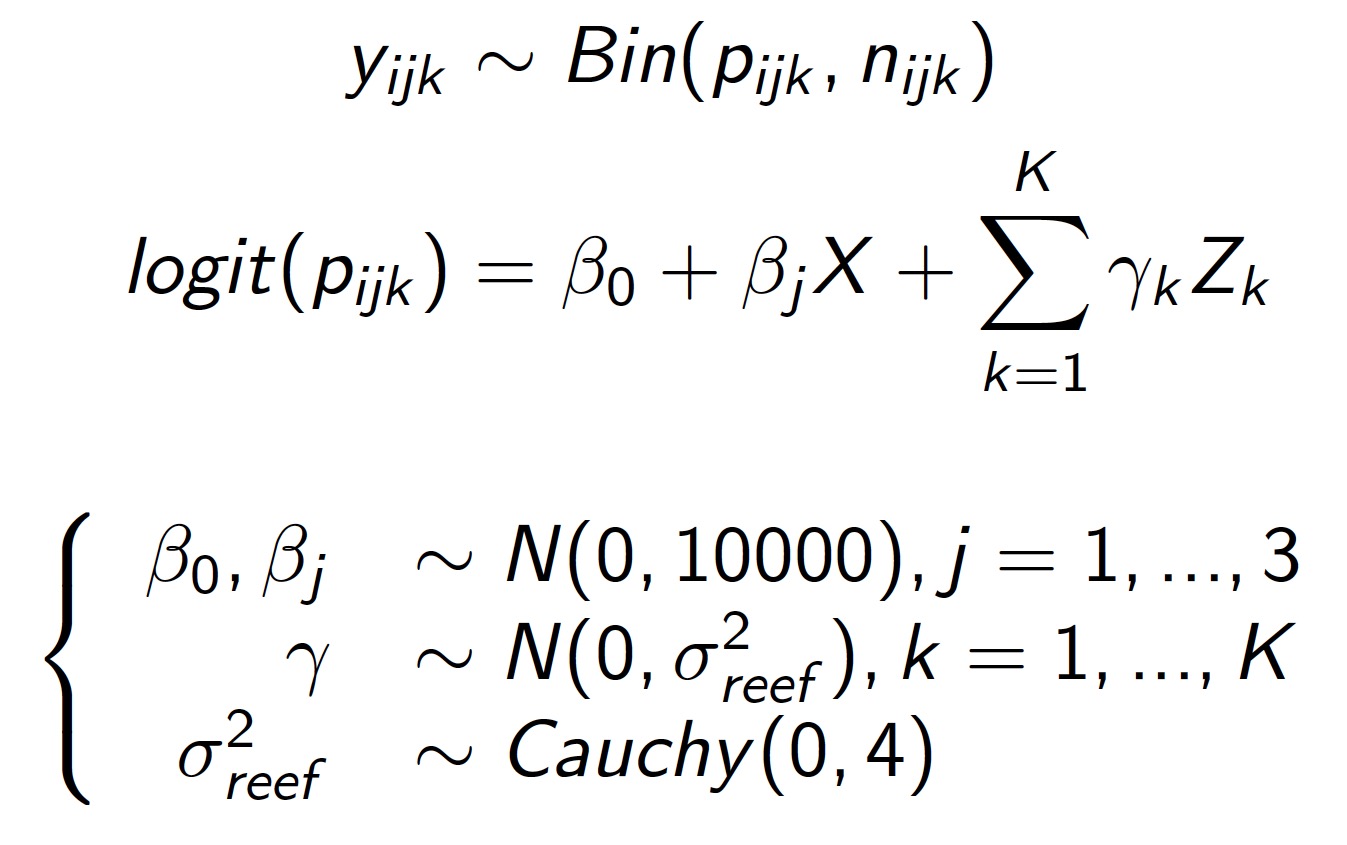 | Beta-binomial: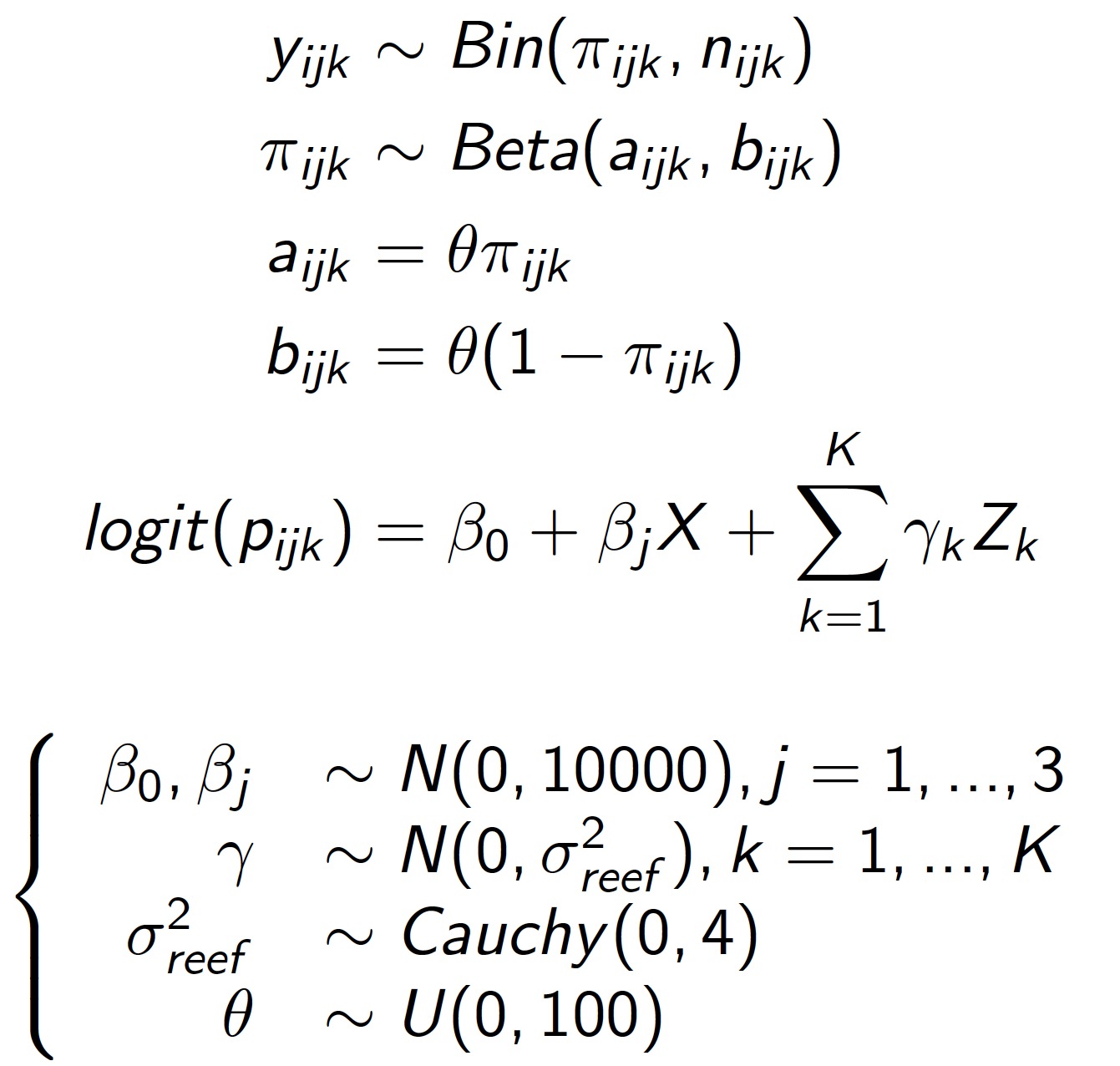 |
| --- | --- |

where β_0_ and β_j_ represent the intercept and effect of the *j*th level of exposure treatment (X), all of which had weakly informative Gaussian priors. γ_k_ represents the effect of the *k*th Reef with priors defined by Gaussian distributions centered around 0 and variance defined by a half-cauchy (scale 4) hyperprior. The dispersion parameter (θ) had a weakly informative Uniform prior (0,100).

Each Bayesian model included 30,000 iterations across three chains with a burn-in of 5,000 and thinning rate of 10 per chain. Pairwise comparisons were derived from the model posteriors. Chain mixing and convergence were assessed via traceplots, autocorrelation and Gelman-Rubin diagnostics (all scale reduction factors less and 1.05). All Bayesian models were fit using JAGS [2] using the R2jags [3] and coda [4] packages for R [1].

**Table S1.** Mean prevalence of coral disease and other indicators of compromised coral health at sites within three sediment plume exposure categories determined by MODIS satellite imagery: low (0 to 9 plume exposure days), moderate (40 to 68 plume exposure days) and high (296 to 347 plume exposure days), and results of Generalized Linear Mixed Model (GLMM) and Bayesian hierarchical linear mixed model analyses. Mean prevalence calculated as the percentage of colonies with disease or compromised health as a percentage of the total number of corals per transect.

|  | **Sediment Plume Exposure Category** | | |  |  |  |  | | | |
| --- | --- | --- | --- | --- | --- | --- | --- | --- | --- | --- |
|  | **Low** | **Moderate** (n = 9 transects) | **High** (n = 9 transects) | **GLMM**  **Probability**  **Bayesian Probability** | | | **Bayesian Probability** | | | |
|  | (n = 18 transects) | (n = 9 transects) | (n = 6 transects) | **Pair-wise Comparisons (*z*, p)**  **Pair-wise Comparisons**  **Pair-wise Comparisons** | | | **Pair-wise Comparisons** | | | |
| **Factor** | mean (SE) prevalence (%) | mean (SE) prevalence (%) | mean (SE) prevalence (%) | **High v. Low** | **High v. Moderate** | **Moderate v. Low** | **High v. Low** | **High v. Moderate** | **Moderate v. Low** |  |
| Total disease | 3.1 (0.6) | 4.7 (1.5) | 7.3 (1.6) | -1.74, 0.08 | -1.12, 0.26 | 0.57, 0.57 | 0.9 | 0.83 | 0.68 |  |
| White syndromes | 2.3 (0.5) | 2.9 (1.2) | 6.7 (1.2) | -1.71, 0.09 | -1.76, 0.08 | -0.31, 0.76 | 0.91 | 0.92 | 0.39 |  |
| Brown band | 0.07 (0.04) | 0.8 (0.4) | 0.09 (0.09) | -0.27, 0.79* | 1.14, 0.25* | 2.01, 0.04* | 0.54 | 0.13 | 0.95 |  |
| Black band | 0.03 (0.02) | 0.5 (0.4) | 0.0 (0.0) | 0.03, 0.98* | 0.03, 0.98* | 1.04, 0.30* | 0.02 | 0.01 | 0.87 |  |
| Skeletal eroding band | 0.7 (0.2) | 0.5 (0.2) | 0.4 (0.3) | 0.63, 0.53 | 0.20, 0.84 | -0.58, 0.56 | 0.3 | 0.42 | 0.36 |  |
| Growth anomalies | 0.0 (0.0) | 0.03 (0.03) | 0.2 (0.2) | 0.00, 1.00* | -0.34, 0.74* | 0.03, 0.98* | 0.98 | 0.61 | 0.98 |  |
| Total compromised health | 6.6 (1.7) | 6.4 (1.1) | 38.3 (23.4) | -3.4, 0.00 | 2.72, 0.01 | 0.44, 0.66 | 0.98 | 0.96 | 0.59 |  |
| Sediment necrosis | 0.5 (0.2) | 0.3 (0.2) | 22.7 (8.5) | -3.65, 0.00 | -3.91, 0.00 | -1.19, 0.23 | 0.99 | 1 | 0.12 |  |
| Bleaching | 1.4 (0.4) | 0.7 (0.2) | 9.5 (3.8) | -3.33, 0.00 | -3.62, 0.00 | -0.83, 0.40 | 0.99 | 0.99 | 0.32 |  |
| Sponge overgrowth | 1.0 (0.3) | 1.2 (0.4) | 2.8 (0.3) | -1.89, 0.06 | -1.66, 0.10 | 0.06, 0.95 | 0.92 | 0.89 | 0.55 |  |
| Pigmentation response | 0.3 (0.1) | 1.6 (0.5) | 2.4 (0.3) | -3.72, 0.00 | -1.00, 0.32 | 3.1, 0.00 | 0.99 | 0.76 | 0.99 |  |
| Red algal overgrowth | 0.9 (0.4) | 1.2 (0.4) | 0.4 (0.3) | 0.44, 0.66 | 1.12, 0.26 | 1.02, 0.31 | 0.36 | 0.19 | 0.79 |  |
| Green algal overgrowth | 2.5 (1.1) | 1.5 (0.8) | 0.5 (0.2) | 1.19, 0.24 | 0.29, 0.77 | -1.03, 0.30 | 0.14 | 0.38 | 0.19 |  |

* Caution is required in interpreting these results due to zero-inflated data

**Figure S1.** Non-metric multidimensional scaling (nMDS) plot visualizing variation in the taxonomic composition (genus-level) of coral assemblages at transects within each sediment plume exposure category: low (0 to 9 plume exposure days; n = 18 transects; white triangles), moderate (40 to 78 plume exposure days; n = 9 transects; grey triangles), and high (296 to 347 plume exposure days; n = 6 transects; black squares).


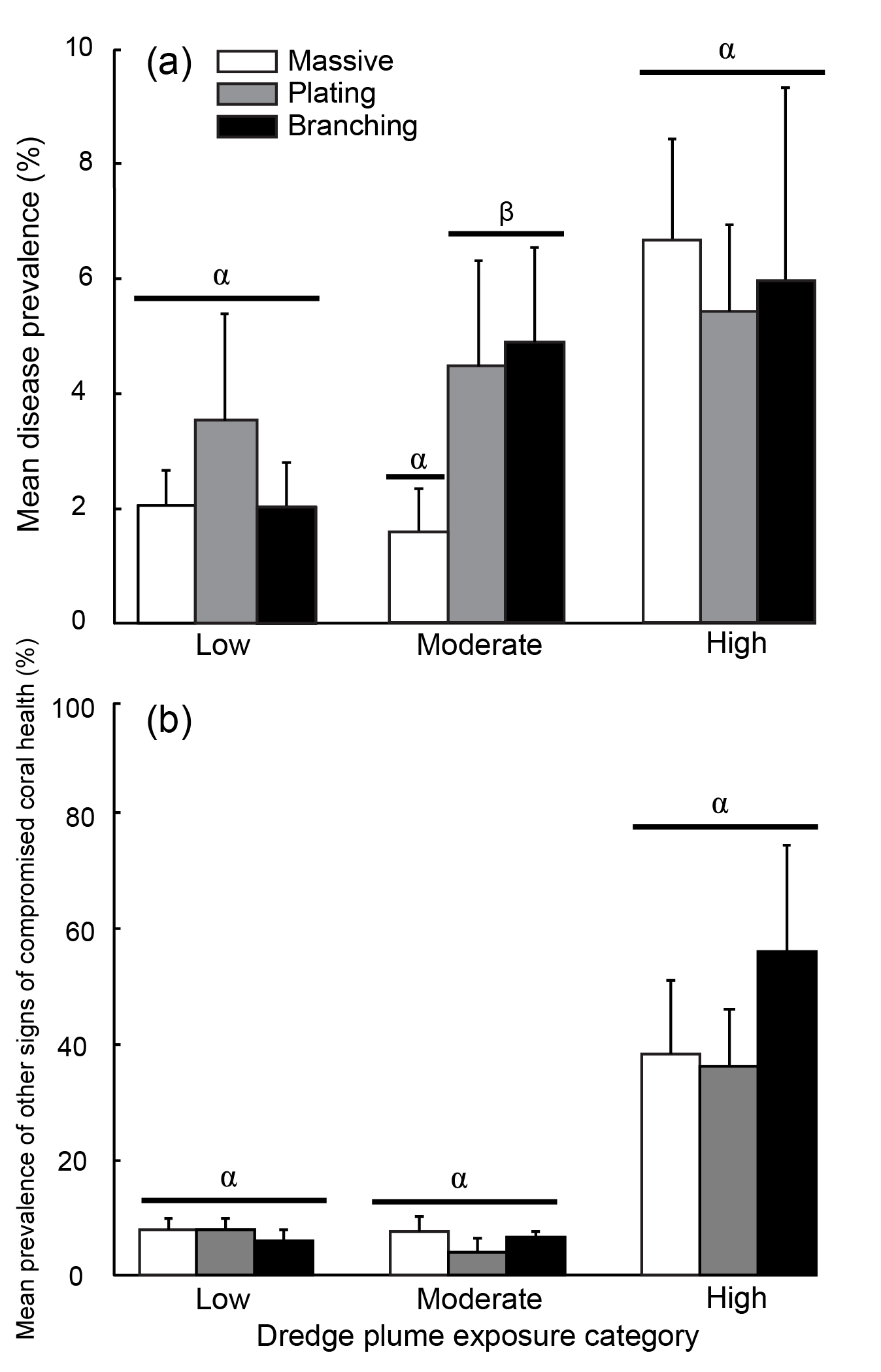


**Figure S2.** Mean prevalence of (a) coral disease and (b) other compromised coral health indicators at sites within three sediment plume exposure categories, low (0 to 9 plume exposure days; n = 15 transects; white bars), moderate (40 to 78 plume exposure days; n = 9 transects; grey bars) and high (296 to 347 plume exposure days; n = 6 transects; black bars), for three coral growth morphologies, massive (n = 1725 colonies), plating (n = 1768 colonies) and branching (n = 3351 colonies). Lettered bars indicate post-hoc groupings (*Tukey’s HSD, p < 0.05*) of morphologies within each exposure category.

**Supplementary References**

1. R Development Core Team. (2015) R: A language and environment for statistical computing. Vienna, Austria: R Foundation for Statistical Computing.
2. Plummer M. (2003) JAGS: A program for analysis of Bayesian graphical models using Gibbs sampling. Proceedings of the 3rd International Workshop on Distributed Statistical Computing. Vienna, Austria: Technische Universit at Wien.
3. Su Y-S, Yajima M. (2012) R2jags: A Package for Running jags from R. R package version 005-07, http://CRANR-project.org/package=R2jags.

4. Plummer M, Best N, Cowles K, Vines K. CODA: Convergence diagnosis and output analysis for MCMC. R News. 2006;6: 7–11.
